# Spatial divergence in the proportions of genes encoding toxic peptide synthesis among populations of the cyanobacterium *Planktothrix* in European lakes

**DOI:** 10.1111/j.1574-6968.2011.02222.x

**Published:** 2011-02-08

**Authors:** Rainer Kurmayer, Eva Schober, Linda Tonk, Petra M Visser, Guntram Christiansen

**Affiliations:** 1 Austrian Academy of Sciences, Institute for LimnologyMondsee, Austria; 2 Institute for Biodiversity and Ecosystem Dynamics, Aquatic Microbiology, University of AmsterdamAmsterdam, The Netherlands

**Keywords:** cyanotoxins, eutrophication, *Planktothrix*, qPCR, spatial isolation, water monitoring

## Abstract

It has been frequently reported that seasonal changes in toxin production by cyanobacteria are due to changes in the proportion of toxic/nontoxic genotypes in parallel to increases or decreases in population density during the seasonal cycle of bloom formation. In order to find out whether there is a relationship between the proportion of genes encoding toxic peptide synthesis and population density of *Planktothrix* spp. we compared the proportion of three gene regions that are indicative of the synthesis of the toxic heptapeptide microcystin (*mcy*B), and the bioactive peptides aeruginoside (*aer*B) and anabaenopeptin (*apn*C) in samples from 23 lakes of five European countries (*n*=153). The *mcy*B, *aer*B, and *apn*C genes occurred in 99%, 99%, and 97% of the samples, respectively, and on average comprised 60 ± 3%, 22 ± 2%, and 54 ± 4% of the total population, respectively. Although the populations differed widely in abundance (10^−3^–10^3^ mm^3^ L^−1^) no dependence of the proportion of the *mcy*B, *aer*B, and *apn*C genes on the density of the total population was found. In contrast populations differed significantly in their average *mcy*B, *aer*B, and *apn*C gene proportions, with no change between prebloom and bloom conditions. These results emphasize stable population-specific differences in *mcy*B, *aer*B, and *apn*C proportions that are independent from seasonal influences.

## Introduction

Water-bloom-forming cyanobacteria threaten humans and livestock due to the production of toxic substances, most prominently the hepatotoxic heptapeptide microcystin. Microcystin and the closely related nodularin have been shown to inhibit eukaryotic protein phosphatases (PP) 1 and 2A. These toxins were responsible for tragedies, for example liver failure and the death of 52 patients in a hemodialysis center in Brazil (Carmichael *et al.*, 2001). In addition to the microcystins, cyanobacteria have been shown to produce many other bioactive peptides (Welker & von Döhren, 2006). For example, aeruginosins are potent inhibitors of serine proteases, such as thrombin and trypsin (Ersmark *et al.*, 2008), anabaenopeptins G, H, and T inhibit carboxypeptidase A (Itou *et al.*, 1999), while the closely related oscillamide Y was shown to inhibit chymotrypsin (Sano & Kaya, 1995) and oscillamide C was shown to be a potent PP1 and 2A inhibitor (Sano *et al.*, 2001). The synthesis of microcystin (MC), aeruginoside (AG), and anabaenopeptin (AP) is encoded by the nonribosomal peptide synthesis (NRPS) pathway (Tillett *et al.*, 2000; Ishida *et al.*, 2007; Rouhiainen *et al.*, 2010;). The interspecific and intraspecific diversity in the production of bioactive nonribosomal peptides is known to be high, i.e. strains or colonies of cyanobacteria such as *Microcystis* sp. or *Planktothrix* spp. isolated from water samples show an impressive diversity in peptide production (Fastner *et al.*, 2001; Welker *et al.*, 2004;).

Quantitative real-time PCR (qPCR) has frequently been used to investigate environmental factors favoring harmful genotypes, i.e. genotypes indicative of the production of the toxic heptapeptide microcystin (Kurmayer & Kutzenberger, 2003). It was hypothesized that the seasonal succession of toxic (microcystin-producing) and nontoxic genotypes is a key mechanism determining microcystin concentrations in surface water (Kardinaal *et al.*, 2007; Hotto *et al.*, 2008;). Several studies reported the occurrence of major changes in the proportion of microcystin genotypes in relation to changes in environmental conditions, for example nitrate concentration (Yoshida *et al.*, 2007), water temperature (Davis *et al.*, 2009), and population density (Briand *et al.*, 2008; Ye *et al.*, 2009;). Some studies reported a decline in the ratio of toxic/nontoxic genotypes in the course of bloom development leading to a transition from an initial high toxicity to low toxicity at maximum cell numbers (Kardinaal *et al.*, 2007; Briand *et al.*, 2009; Bozarth *et al.*, 2010;). Correspondingly, the analysis of clone libraries of variable ribosomal gene regions (Yoshida *et al.*, 2005; Briand *et al.*, 2009;) or the intergenic spacer of the phycocyanin operon (Bozarth *et al.*, 2010) revealed significant changes in genotype composition that might explain the negative correlation between toxic genotype proportion and population density.

It is of interest to find out whether there is a general relationship between population density and the proportion of toxic genotypes or whether the toxic/nontoxic genotype ratio is influenced indirectly by seasonal shifts in genotype composition. In the latter case, it would be concluded that ecophysiological characters other than the ability to produce microcystin are selected for during bloom development. In order to quantify genes encoding microcystin, aeruginoside, and anabaenopeptin synthesis over a wide range in population abundance populations of the filamentous cyanobacterium *Planktothrix* were sampled from 23 lakes in Austria, Germany, the Netherlands, Denmark, and Norway. *Planktothrix* spp. reaches maximum cell numbers during summer, either in stratified layers of the water column (red-pigmented *Planktothrix* occurring in deep lakes of the Alps or in reservoirs) or in shallow turbid lakes (green-pigmented *Planktothrix*). Both ecotypes of *Planktothrix* are known to be highly adapted to low-light conditions and considered separate species (Suda *et al.*, 2002).

## Materials and methods

### Sampling

During 2003 and 2004 four lakes, i.e. Lake Mondsee and Irrsee (Austria), Lake Wannsee (Germany), and Frederiksborg Slotssø (Denmark), were sampled monthly in the course of the EU project PEPCY (http://www.pepcy.de). Another 10 lakes were sampled at least three times a year during the growth season and another nine lakes were sampled once (Table 1). In addition, phytoplankton sampled monthly from Lake Wannsee during 1999 and 2000 was included (Kurmayer *et al.*, 2003). Shallow polymictic lakes were sampled by collecting and combining 1 L each meter through the water column at maximum depth (e.g. Lake Wannsee and Frederiksborg Slotssø). Deep physically stratified lakes were sampled by combining 1 L from every 3 m to a depth of 20 m (e.g. Lake Mondsee, Wörthersee). Depending on the density of phytoplankton, 0.2–2 L were filtered onto glass fiber filters (Whatman GF/C) using low-vacuum filtration and filters were stored at −20 °C. DNA was extracted from the filters using the standard phenol–chloroform procedure (Kurmayer *et al.*, 2003). Subsamples were Lugol fixed and analyzed for phytoplankton composition using the inverted microscope technique (Wetzel & Likens, 2000). The detection limit of *Planktothrix* spp. in the inverted microscope was one trichome per sedimentation chamber (50 mL volume). *Planktothrix* spp. was identified according to the morphological criteria described in Komárek (2003).

**Table 1:** Origin of water samples

Lake	Country	Latitude (°N)	Longitude (°E)	Surface area (km^2^)	Maximum depth (m)	Number of samples	Sampling period	Pigmentation of Planktothrix*	Abundance of *Planktothrix*†	Trophy
Intensively sampled lakes										
Frederiksborg Slotssø	DK	55°56′	12°18′	0.2	9	14	Jul 03–Sep 04	Green	A	Eutrophic
Irrsee	AT	47°55′	13°18′	3.5	32	20	Apr 03–Dec 04	Red	A	Mesotrophic
Mondsee	AT	47°49′	13°22′	13.8	68.3	27	Apr 03 –Dec 04	Red	A	Mesotrophic
Wannsee	DE	52°25′	13°10′	2.7	10	27	Jun 99–Oct 00 Aug 03–Dec 03	Green	A	Eutrophic
Occasionally sampled lakes										
Joppe	NL	52°02′	04°50′	0.9	42	12	Jul 03–Aug 05	Green	R	Eutrophic
Klinkenberger Plas	NL	52°04′	05°02′	0.3	35	4	May 04–Aug 04	Red/green	A	Eutrophic
Offensee	AT	47°45′	13°50′	0.6	38	4	Jul 03 Jul 04	Red	A	Mesotrophic
Schwarzensee	AT	47°45′	13°30′	0.5	54	6	Apr 03–Oct 04	Red	R	Oligotrophic
Slotermeer	NL	52°09′	05°63′	12.4	6	3	May 04–Oct 04	Green	A	Eutrophic
Steinsfjorden	NO	60°05′	10°19′	13.9	24	8	Jun 03–Aug 04	Red/green	A	Mesotrophic
Tjeukemeer	NL	52°05′	05°50′	20	5	3	May 04–Oct 04	Green	A	Eutrophic
Wolfgangsee	AT	47°45′	13°25′	12.8	113	5	Jun 03–Nov 04	Red	R	Oligotrophic
Wörthersee	AT	46°37′	14°07′	19.4	85.2	4	Aug 03–Nov 04	Red	D	Mesotrophic
Zegerplas	NL	52°13′	04°67′	0.7	34	7	Jun 03–Aug 05	Green	R	Eutrophic
Lakes sampled once										
Albufera Lagoon	ES	39°20′	0°21′W	21	3	1	Aug 04	Green	A	Eutrophic
Hallwilersee	CH	47°17′	08°12′	10	48	1	Sep 04	Red	D	Mesotrophic
Havel (Potsdam)	DE	52°45′	12°10′	-	4	1	Sep 04	Green	A	Eutrophic
Lago Maggiore	IT	45°28′	08°40′	212	372	1	Sep 04	Red	A	Mesotrophic
Miedwie	PL	53°17′	14°54′	35.5	44	1	Sep 04	Red	A	Mesotrophic
Pozillo, Sicily	IT	37°39′	14°35′	7.7	55	1	Jan 06	Red	D	Eutrophic
Prizzi, Sicily	IT	37°43′	13°24′	1.3	44	1	Feb 06	Red	D	Eutrophic
Sapanca	TR	40°69′	30°27′	46.8	55	1	Sep 04	Red	A	Mesotrophic
Talsperre Weida	DE	50°42′	11°58′	0.9	22.5	1	Sep 04	Red	A	Mesotrophic

* Samples were grouped according to the visual inspection in the microscope: (1) red-pigmented; (2) green-pigmented; (3) red/green-pigmented.

† A, abundant (<50%); R, rare (<10%); D, dominant (>50% in numbers according to the inspection in the microscope).

### Design of the primers and probes

The TaqMan assay (TNA) was used to quantify (1) the intergenic spacer region of the phycocyanin operon (PC-IGS) to estimate the total population of *Planktothrix*, (2) the *mcy*BA1 gene encoding the first adenylation domain of the *mcy*B gene that is indicative of all *Planktothrix* cells containing the *mcy* gene cluster (Christiansen *et al.*, 2003), (3) the *aer*B gene coding for an epimerase domain of the aeruginoside gene cluster in *Planktothrix* (Ishida *et al.*, 2007), and (4) the *apn*C gene encoding the *N*-methyl transferase part of the *apn*CA2 adenylation domain that is involved in anabaenopeptin synthesis (Rouhiainen *et al.*, 2010; R. Kurmayer and G. Christiansen, unpublished results).

Primers and the TaqMan probe that were specifically bound to PC-IGS or *mcy*BA1 of *Planktothrix* have been described previously (Schober & Kurmayer, 2006; Ostermaier & Kurmayer, 2009;). Although the *mcy*BA1 region was found to be, in part, affected by recombination events, a conserved region was identified based on 27 strains sequenced for the whole *mcy*BA1 domain previously (AJ890255–AJ890282; Kurmayer & Gumpenberger, 2006). For the design of the primers and probes to quantify the *aer*B gene coding for an epimerase domain of the aeruginoside gene cluster (Ishida *et al.*, 2007) a 343-bp region was amplified and sequenced from 25 *Planktothrix* strains (GQ917083–GQ917107). From these sequences, the primers and probe were designed to amplify 89 bp of *aer*B (strain CCAP1459/36 had one substitution) using the primer express 2.0 software (Applied Biosystems, Vienna, Austria, Supporting Information, Tables S1, S2). In addition, the region encoding the *N*-methyl transferase part of the *apn*CA2 adenylation domain that is involved in anabaenopeptin synthesis (Rouhiainen *et al.*, 2010) was amplified and sequenced from thirty strains (GQ917053–GQ917082). The primers and probe were designed from a region with a minimum sequence variation (142 bp): three strains (CYA126/8, CCAP1459/14, CCAP1459/31) showed a single-point mutation. The probes were labelled with a fluorescent reporter dye that was covalently attached to the 5′-end (FAM, 6-carboxyfluorescein) and a fluorescent quencher dye attached to the 3′-end (TAMRA, 6-carboxytetramethylrhodamine). Concentrations of the primers and probes were optimized according to the manufacturer's instructions (ABI TaqMan Universal PCR MasterMix, Table S1).

### Quantitative real-time PCR (qPCR)

qPCR analysis was performed using the geneamp5700 as described (Kurmayer & Kutzenberger, 2003). Each sample was analyzed by four independent qPCRs set up from the same DNA extract. To establish the calibration curves, a dilution series of predetermined DNA concentrations from the extracts of *Planktothrix* strain PCC7821 (carrying the *mcy*B, *aer*B, and *apn*C gene) was prepared and the DNA content in the template (expressed in biovolume) was related to the threshold cycle (*C*_t_) value (defined as the *C*_t_ to reach a manually set fluorescence) from 0.55 × 10^−8^ mm^3^ (4.1 cells)−0.55 × 10^−5^ mm^3^ (4100 cells) per template (Table 2). Proportions of *mcy*B, *aer*B, and *apn*C genes were calculated from the biovolume of the total population as determined via the PC-IGS region by qPCR from the same DNA extract. Calibration curves were not extrapolated beyond the highest dilution, which was defined arbitrarily as the limit of quantification corresponding to 0.55 × 10^−8^ mm^3^ in the template. The specificity and robustness of each TNA was tested by adding DNA originating from other organisms as a background. DNA extracted from the *Microcystis* strains HUB524, HUB53, and *Synechococcus* strain MW15#2SUB was added to the DNA of strain PCC7821 in two concentrations, 1.7 × 10^4^ and 1.7 × 10^5^ cells per template consisting of 13% of strain HUB53, 11% of strain HUB524, and 76% of strain MW15#2SUB, respectively. The addition of the background did not influence the *C*_t_ value for strain PCC7821 in the range of 0.55 × 10^−8^–0.55 × 10^−5^ mm^3^. All results on *mcy*B, *aer*B, and *apn*C abundance were compared using a nonparametric test (Kruskal—Wallis one way anova on Ranks). If an overall significant difference between lake groups was found (*P*<0.05), pair-wise *post hoc* comparisons were performed using the Dunn test (sigma plot for Windows Version 11.0).

**Table 2:** Calibration curves used for qPCR in the present study

qPCR	Gene region coding for	Calibration curve	*E* (%)*	*R*^2^	*n*	*P*
PC-IGS	Intergenic spacer region of the phycocyanin operon	*y*=−3.895*x*+9.149	81	0.997	4	< 0.001
Pl *mcy*BA1	First adenylation domain of the *mcy*B gene	*y*=−3.520*x*+11.372	92	0.995	4	< 0.001
Pl aerug	Epimerase of the *aer*B gene	*y=*−3.316*x*+11.601	100	0.982	4	< 0.001
Pl NMT	*N*-methyl transferase of the *apn*C gene	*y=*−4.032*x*+15.045	77	0.936	4	< 0.001

* The amplification efficiency (*E*) was calculated as follows: *E*=(10^−1/slope^−1) × 100.

*y*, the number of PCR cycles at the set fluorescence threshold level (*C*_t_-value at 0.1); *x*, the amount of DNA in the template (as log 10 mm^3^ of biovolume).

## Results

### Biovolume of *Planktothrix* spp

The phytoplankton consisted of cyanobacteria, diatoms, chrysophytes, cryptophytes, dinophytes, and green algae. Average biovolume of the total phytoplankton differed according to the lakes' trophic state, i.e. the lakes located in the Alps and Lake Steinsfjorden in Norway were oligotrophic to mesotrophic, while lakes located in Denmark, Germany, and the Netherlands were classified as eutrophic (Table 1). In general, *Planktothrix* biovolume varied from 10^−3^ to 10^3^ mm^3^ L^−1^ (*n*=146). In seven samples, no *Planktothrix* either by qPCR or by the microscope was detected. There was a clear linear correlation between microscopical detection and detection of *Planktothrix* by qPCR: *y*=0.909*x*−0.441 (*R*^2^=0.72; *P*<0.0001; *n*=101), where *y* was log_10_ biovolume as determined by the qPCR and *x* was log_10_ biovolume as determined by the microscope (Fig. S1). Surprisingly for samples from Lake Offensee (Austria) and Talsperre Weida (Germany), only irregular amplification curves by the PC-IGS assay were obtained. As these amplification curves made an estimate of the total population density impossible, these five samples were excluded from further analysis.

According to the pigmentation of *Planktothrix*, the samples were assigned to the following groups: (1) samples obtained from red-pigmented populations including lakes located within the Alps (*n*=65), for example Lake Mondsee, Lake Irrsee, and reservoirs (Pozzillo, Prizzi, Sapanca); (2) samples obtained from green-pigmented populations typically occurring in shallow eutrophic lakes (*n*=64), for example Lake Wannsee and Frederiksborg Slotssø; and (3) samples obtained from red/green-pigmented populations (*n*=12), for example Lake Steinsfjorden, Klinkenberger Plas (Table 1). Within the red or green pigmentation type, the samples were assigned to three categories according to population density: (1) dense populations: >1.0 mm^3^ L^−1^, minimum–median–maximum=1.1–2.7–903 mm^3^ L^−1^, *n*=6 (red), *n*=21 (green), *n*=4 (mixed); (2) moderately dense populations: 0.1–1.0 mm^3^ L^−1^, minimum–median–maximum=0.1–0.28–0.92, *n*=17 (red), *n*=16 (green), *n*=3 (mixed); and (3) sparse populations: <0.1 mm^3^ L^−1^, minimum–median–maximum=2.6 × 10^−3^–0.03–0.09, *n*=42 (red), *n*=27 (green), *n*=5 (mixed).

### Abundances of the *mcy*B, *aer*B, and *apn*C genes

The *mcy*B, *aer*B, and *apn*C genes occurred in 99%, 99%, and 97% of all samples (*n*=119) that contained *Planktothrix* spp. above the quantification threshold (0.55 × 10^−8^ mm^3^ or 4.1 cells in the template). Below the quantification threshold of *Planktothrix*, the results were more variable and *mcy*B, *aer*B, and *apn*C genes occurred only in 96%, 19%, and 7% of the samples (*n*=27), respectively. It is likely that this lower frequency of *aer*B and *apn*C resulted from the lower sensitivity of the respective qPCR assays when compared with the qPCR assay for *mcy*B (Table 2).

**Fig. 1:**
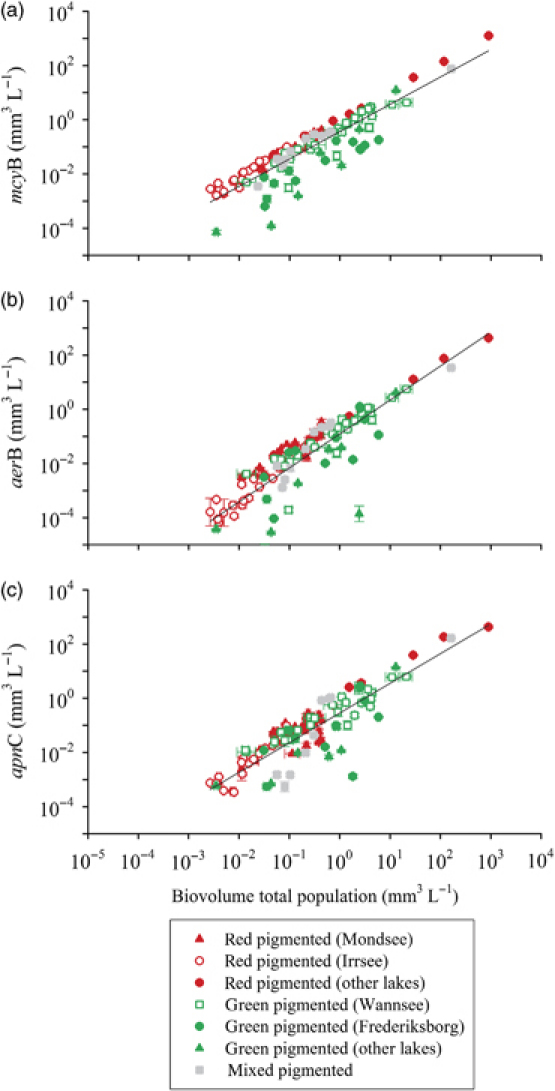
Relationship between *Planktothrix* spp. abundance and (a) the *mcy*B (microcystin) gene abundance, (b) the *aer*B (aeruginoside) gene abundance, and (c) the *apn*C (anabaenopeptin) gene abundance (mean ± 1 SE mm^3^ of biovolume L^−1^). For details on the regression curves see text.

Notably, the abundance of all three *mcy*B, *aer*B, and *apn*C genes was linearly correlated to the total *Planktothrix* biovolume as estimated by qPCR: for *mcy*B the linear regression curve was *y*=1.010*x*−0.441 (*R*^2^=0.78, *n*=133), for *aer*B the regression curve was *y*=1.253*x*−0.910 (*R*^2^=0.71, *n*=123), and for the *apn*C the regression curve was *y*=1.095*x*−0.545 (*R*^2^=0.76, *n*=122), where *y* was the log_10_ biovolume of the respective gene region and *x* was the log_10_ biovolume of the total population as estimated via the PC-IGS region by qPCR (Fig. 1).

### Proportions of the *mcy*B, *aer*B, and *apn*C genes

**Fig. 2:**
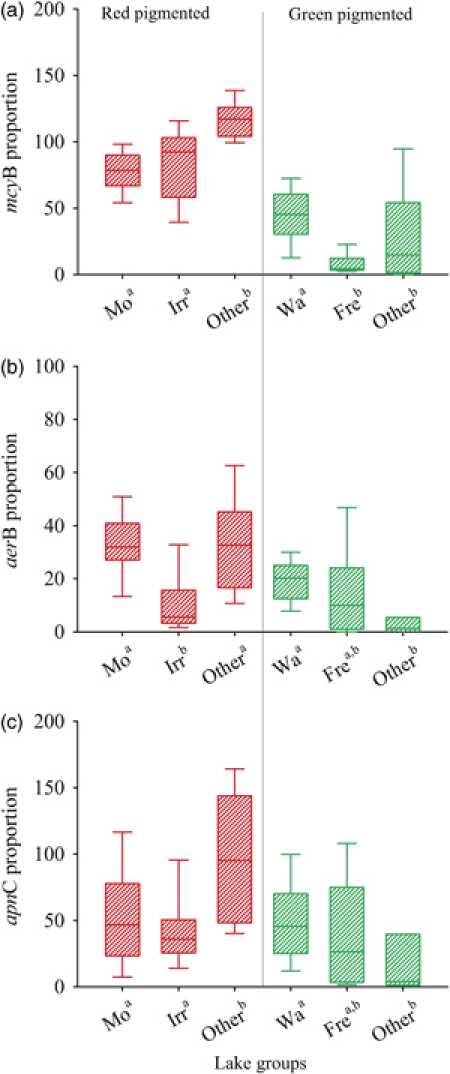
Proportions (%) of (a) *mcy*B, (b) *aer*B, and (c) *apn*C among red- or green-pigmented *Planktothrix* spp. Mo, Lake Mondsee (Austria); Irr, Lake Irrsee; Wa, Lake Wannsee; Fre, Frederiksborg Slotssø; other, occasionally sampled lakes and lakes sampled only once. The whiskers of each box indicate the 10th and 90th percentiles. If an overall difference between lake groups within a pigmentation type was found, superscripts (a, b) indicate homogeneous subgroups not significantly different at *P*<0.05.

**Fig. 3:**
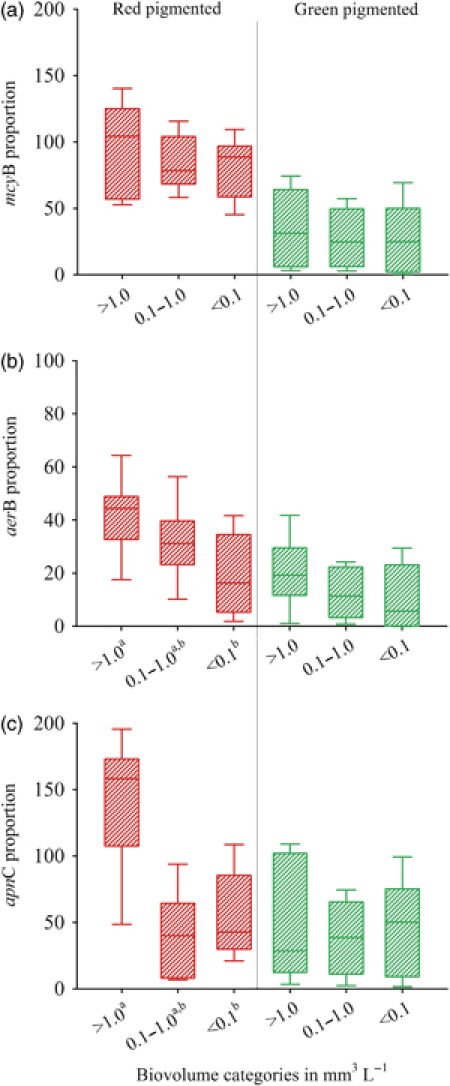
Proportions (%) of (a) *mcy*B, (b) *aer*B, and (c) *apn*C within three different population density categories of red- or green-pigmented *Planktothrix* spp.: dense,>1.0 mm^3^ L^−1^; moderately dense, 0.1–1.0 mm^3^ L^−1^; sparse,<0.1 mm^3^ L^−1^. The whiskers of each box indicate the 10th and 90th percentiles. If an overall difference between lake groups within a pigmentation type was found, superscripts (a, b) indicate homogeneous subgroups not significantly different at *P*<0.05.

The average proportions of the *mcy*B, *aer*B, and *apn*C genes were 60 ± 3%, 22 ± 2%, and 54 ± 4% respectively. On average the proportions of all three *mcy*B, *aer*B, and *apn*C genes were significantly higher among red-pigmented when compared with all the green-pigmented populations, i.e. red-pigmented: *mcy*B: 86 ± 3% (*n*=57); *aer*B: 26 ± 2% (*n*=55); *apn*C: 60 ± 6% (*n*=52) vs. green-pigmented: *mcy*B: 31 ± 4% (*n*=48); *aer*B: 16 ± 2% (*n*=46); *apn*C: 43 ± 5% (*n*=43), (Mann–Whitney rank-sum test, *mcy*B, *aer*B: *P*≤0.001, *apn*C: *P*=0.059, Figs 2 and 3). Within each pigmentation type, the *mcy*B proportion differed spatially between populations, for example, it was higher among samples from Lake Wannsee (43.0 ± 4%, *n*=26) than among samples from Lake Frederiksborg Slotssø (8 ± 2%, *n*=13, *P*<0.001, Fig. 2a). Analogously, the average *aer*B proportion differed significantly not only between red/green-pigmented populations, but also within a pigmentation type (*P*<0.001). When compared with Lake Mondsee (34 ± 3%, *n*=27) the *aer*B proportion in Lake Irrsee was significantly reduced (11 ± 2%, *n*=18), while the *aer*B proportion in Lake Wannsee (19 ± 2%, *n*=26) differed significantly from all other green-pigmented samples (6 ± 4%, *n*=7, Fig. 2b). Although less pronounced, a spatial difference was also found for the average *apnC* proportion within red/green-pigmented populations (*P*<0.001, Fig. 2c). It is concluded that *Planktothrix* populations differed in their average *mcy*B, *aer*B, and *apn*C proportion, which is not restricted to the difference between the red- or green-pigmented groups, but also occurs within the same pigmentation type.

**Fig. 4:**
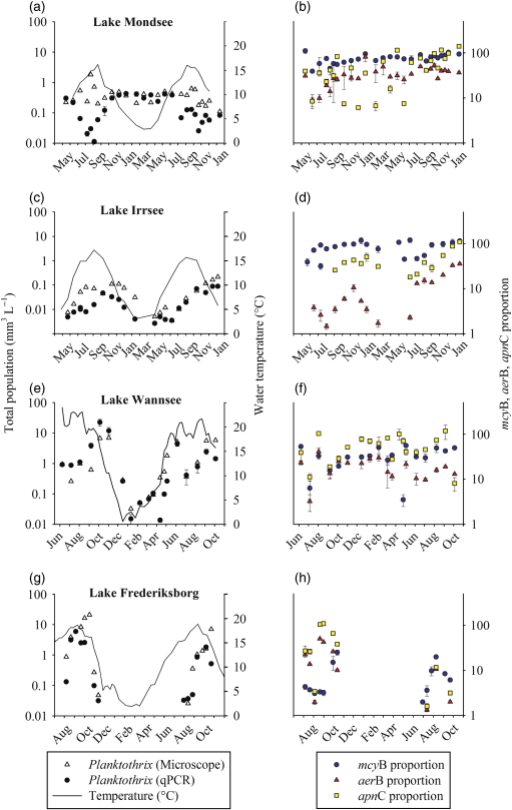
Total population density of *Planktothrix* spp. as counted in the microscope and estimated via qPCR and seasonal variability in the *mcy*B, *aer*B, and *apn*C proportion (mean ± SE in percent), in (a, b) Lake Mondsee, (c, d) Lake Irrsee, (e, f) Lake Wannsee, (g, h), and Lake Frederiksborg Slotssø. The straight line indicates water temperature integrated either from 0 to 20 m (Lake Mondsee, Lake Irrsee) or the total water column.

In contrast to the spatial differences, the *mcy*B, *aer*B, and *apn*C proportions varied independently between dense (>1.0 mm^3^ L^−1^), moderately dense (0.1–1.0 mm^3^ L^−1^), and sparse (<0.1 mm^3^ L^−1^) population density categories among green-pigmented populations (*P*>0.05, Fig. 3). Among red-pigmented populations, the *aer*B and *apn*C proportion of the dense populations differed significantly from the moderately dense and sparse density categories (*P*<0.002, Fig. 3b and c). However, a Pearson product–moment correlation analysis revealed no relationship between *mcy*B, *aer*B, or *apn*C proportions and total population density within either green- or red-pigmented populations (*R*<0.5). Besides the variation in population abundance between populations, the total *Planktothrix* biovolume varied seasonally within lakes, i.e. 38-, 33-, 1650-, and 189-fold in Lake Mondsee, Lake Irrsee, Lake Wannsee, and Lake Frederiksborg Slotssø, respectively (Fig. 4). The Pearson product–moment correlation analysis did not reveal any significant relation between the *mcy*B proportion and total population density in all four lakes (*R*<0.5) and also no relationship between *aer*B and *apn*C proportion and population density in Lake Mondsee, Wannsee, and Frederiksborg Slotssø. Only among the samples obtained from Lake Irrsee a positive correlation between *aer*B and *apn*C proportion and the total population density was found (*aer*B: *R*=0.78; *apn*C: *R*=0.82). It is concluded that, within a pigmentation type, the *mcy*B, *aer*B, and *apn*C proportions were not related to population density between lakes and in the majority of samples also not within lakes during seasonal succession.

## Discussion

### Estimation of *mcy*B, *aer*B, and *apn*C gene abundance by qPCR

On a genetic level, recombination affecting particular gene regions can lead to an over- or underestimation of proportions of particular gene regions in a population. Both for PC-IGS and *mcy*BA1, recombination events have been described (e.g. Manen & Falquet, 2002; Kurmayer & Gumpenberger, 2006;). While for a particular gene region the influence of recombination can never be excluded, this potential bias has been minimized by sequencing the amplified gene regions in a larger number of strains isolated from nine European countries and 28 water bodies (Table S2). These strains did not show signs of past gene conversion (recombination) events. However, it cannot be excluded that the occurrence of single base pair substitutions within the amplified PC-IGS region resulted in the *mcy*B and *apn*C proportions over 100% as observed for the most dense populations of red-pigmented populations (>1.0 mm^3^ L^−1^, Fig. 3). It also seems likely that recombination events caused the irregular amplification curves of the PC-IGS assay as observed for samples from Lake Offensee (Austria) and Talsperre Weida (Germany). Two alternative qPCR reference gene assays to quantify the total population targeting either the 16S rRNA gene or the 16S rRNA gene internal transcribed spacer region were used to control the estimates by the PC-IGS region for the whole data set (data not shown). While both rRNA gene assays gave useful PCR amplification curves for the samples from Lake Offensee and Talsperre Weida, the application of these alternative qPCR assays did not eliminate the *mcy*B and *apn*C proportions over 100%. The three different reference gene assays were highly significantly related (*R*≥0.96, *n*=146); however, the PC-IGS assay was found to have the highest correlation coefficient in explaining the variation among *Planktothrix* biovolume as detected in the microscope (Fig. S1). It has been argued during the reviewing process of this paper that the *mcy*B, *aer*B, and *apn*C proportions should be related to one and the same reference gene assay. Nevertheless, it seems reasonable that combining several gene probes to amplify both the total population, as well as specific genes encoding toxin synthesis, will control the influence of unknown recombination events and therefore contribute to the development of a more robust qPCR protocol.

### Abundances of the *mcy*B, *aer*B, and *apn*C genes

The results of this study imply that among European *Planktothrix* populations genes encoding toxin synthesis occurred frequently and varied independently in their proportion from the total population density over a wide range in population abundance as well as ecosystems that differ in trophy and morphometry, such as water depth (shallow lakes vs. deep reservoirs and deep lakes in the Alps). Indeed the abundance of the total population was a strong predictor of *mcy*B, *aer*B, and *apn*C abundance, implying that those populations containing such a gene are unlikely to lose it in the short term and become nontoxic in the course of seasonal development (Fig. 1). These results are relevant for our understanding of the likelihood of the occurrence of toxic algal blooms under specific environmental conditions.

Although genetic methods are only able to indicate the potential of toxic peptide synthesis, it has been shown previously that gene numbers estimated by qPCR can be used to infer concentrations of different peptide classes such as the nodularins (Koskenniemi *et al.*, 2007) or the microcystins (Hotto *et al.*, 2008; Okello *et al.*, 2010; Ostermaier & Kurmayer, 2010;). On the other hand chemical methods such as MALDI-TOF MS or LC-MS typically have a much higher resolution and resolve a large number of individual peptide structural variants (Welker & Erhard, 2007; Rohrlack *et al.*, 2008;). The drawback of the chemical methods is the requirement for purified standard substances in order to estimate individual peptide concentrations (e.g. Halstvedt *et al.*, 2008). During this study's period, peptide concentrations have been determined by LC-MS for samples obtained from Lake Mondsee and Lake Irrsee on a relative scale (using MC-LR as an internal standard, R. Kurmayer, T. Rohrlack, NIVA, unpublished data). It is interesting to note that when related to *Planktothrix* biovolume, the peptide aeruginosin A (*m*/*z* 617) was found on average fourfold more abundant in samples from Lake Mondsee (*n*=40) when compared with samples obtained from Lake Irrsee (*n*=18). In contrast, the same ratio calculated for the sum of MC-RR (*m*/*z* 1024) and MC-LR (*m*/*z* 981) as well as the sum of AP-B (*m*/*z* 837) and AP-F (*m*/*z* 851) showed no difference between the two lakes. This difference in peptide concentrations corresponds with the *mcy*B, *aer*B, and *apn*C proportions reported in those two lakes (Figs 2 and 4). It is concluded that gene numbers and proportions estimated by qPCR can be used to estimate the population-specific production of bioactive and toxic peptides such as aeruginosides and anabaenopeptins in water bodies.

While *Planktothrix* populations showed relatively minor changes in their average toxic genotype proportion between prebloom and bloom conditions, the spatial divergence in the *mcy*B, *aer*B, and *apn*C proportion between populations was pronounced. This implies that although variation in the *mcy*B proportion within the same population during the season was recorded, the measurements on the *mcy*B ratio between populations were consistently different (Fig. 2). For example, *Planktothrix* in Lake Wannsee (Germany) not only never contained *mcy*B in all cells (as observed among red-pigmented populations) but also never contained such a low percentage of *mcy*B as observed in the samples of Lake Frederiksborg Slotssø (Denmark). We reported previously that the individual *Planktothrix* populations differed significantly in the occurrence of specific *mcy*B genotypes over several years (Kurmayer & Gumpenberger, 2006). Correspondingly, a pronounced spatial divergence in the proportion of the microcystin genotype has been observed for six spatially isolated populations of *Microcystis* sampled during 1 year in Uganda, East Africa (Okello *et al.*, 2010). In a similar manner, Sabart *et al.* (2010) reported spatial differences in the microcystin genotype proportion of *Microcystis* sampled from several interconnected sites along the Loire River in France. The authors concluded that the relative selection of the microcystin-producing and non-microcystin-producing genotype occurs at the scale of each ecosystem and depends on many local environmental factors and processes. It is likely that as opposed to a single environmental factor, the proportion of *mcy*B, *aer*B, and *apn*C genes is influenced by multiple factors, both on a spatial scale and in the course of seasonal succession. Our conclusion is important in the light of experimental studies that attempt to find environmental factors that regulate the proportion of toxic genotypes in populations of toxic cyanobacteria such as *Planktothrix* or *Microcystis*. It has been shown earlier that nontoxic and toxic strains have diversified by factors not directly linked to microcystin production (Tanabe *et al.*, 2007; Christiansen *et al.*, 2008;). Probably, the benefits/costs solely attributable to microcystin, aeruginoside, and anabaenopeptin synthesis under varying population density conditions can only be estimated by comparing the growth between a generated mutant deficient in microcystin, aeruginoside, and anabaenopeptin production vs. the wildtype strain (Hesse *et al.*, 2001). In the future, it would be required to perform competition experiments between *Planktothrix* strains and specific mutants under varying growth-limiting conditions in order to estimate the extent of benefits/costs of either of the three peptide classes on the cellular growth rate.

## Statement

Re-use of this article is permitted in accordance with the Terms and Conditions set out at: http://wileyonlinelibrary.com/onlineopen#OnlineOpen-Terms

## Supporting information

**Fig. S1.** Comparison of the total *Planktothrix* spp. biovolume (mm^3^ L^−1^) estimated from lake water samples (Table 1) by counting under the microscope and by qPCR, mean ±1 SE.

**Table S1.** Oligonucleotides used for PCR and sequencing, and primers and probes used for qPCR to quantify six genotypes of *Planktothrix* spp.

**Table S2.** *Planktothrix* strains used in this study and sequences used to design TaqMan probes for quantitative real-time PCR.
