# Supplementary material for: Spatial divergence in the proportions of genes encoding toxic peptide synthesis among populations of the cyanobacterium Planktothrix in European lakes
Source: FEMS Microbiol Lett. 2011 Feb 8;317(2):127–37. doi: 10.1111/j.1574-6968.2011.02222.x (PMC3118798; doi:10.1111/j.1574-6968.2011.02222.x)
Supplement: Supplementary file 1 [file fml0317-0127-SD1.doc]

**Supporting Information**

Primers (Supp. Table 1), sequenced strains (Suppl. Table 2) and correlation observed between the microscope and qPCR (Supp. Fig. 1).

Suppl. Table 1. Oligonucleotides used for PCR and sequencing, and primers and probes used for qPCR to quantify six genotypes of *Planktothrix* spp.

| PCR assay | Gene region coding for | | Fwd primer (5´-3´) | nM^1^ | TaqMan probe (5´-3´) | nM^1^ | Rev primer (5´-3´) | nM | Amplicon length (bp) | Annealing (°C) |
| --- | --- | --- | --- | --- | --- | --- | --- | --- | --- | --- |
| **PCR for sequencing** | |  |  |  |  |  |  |  |  |  |
| *aer*B | Epimerase of the *aer*B gene | | acccgacccgatcactttaat | 500 | n/a |  | gcccaatgtttaaaggagctagttt | 500 | 343 | 54 |
| *apn*C | N-methyl transferase of the *apn*C gene | | CGTGCAGATGATGACCTATCCA | 500 | n/a |  | AAGGTTCGCAATACTTCAGGGTT | 500 | 470 | 55 |
| **qPCR** | |  |  |  |  |  |  |  |  |  |
| PC-IGS^2^ | Intergenic spacer region of the phycocyanin operon | | GAGCAGCACTGAAATCCAAG | 50 | TTTGGCTTGACGGAAACGACCAA | 100 | GCTTTGGCTGCTTCTAAACC | 300 | 72 | 60 |
| Pl *mcy*B^3^ | First adenylation domain of the *mcy*B gene | | ATTGCCGTTATCTCAAGCGAG | 900 | TTTTTGTGGAGGTGAAGCTCTTTCCTCTGA | 100 | TGCTGAAAAAACTGCTGCATTAA | 900 | 76 | 60 |
| Pl aerug | Epimerase of the *aer*B gene | | TGTCTTCGGGTGAACAA | 900 | ACCACAATGGCAGAAATTTCAGA | 150 | CGAAATTAAGGGGCCTT | 900 | 89 | 55 |
| Pl NMT | N-methyl transferase of the *apn*C gene | | TCCAAAATATTTTAAACACTCAAG | 900 | TgAAAATATTCCCAATATTCgTCTgA | 250 | CTTAAAATAGCTTTTAATTGTTTAATA | 900 | 142 | 55 |

^1^ final concentration in nM, ^2^Schober & Kurmayer (2006), ^3^Ostermaier & Kurmayer (2010)

Suppl. Table 2: *Planktothrix* strains used in this study and sequences used to design TaqMan probes for quantitative real time PCR. EMBL Accession numbers are from: AJ558134 - AJ558160 (Kurmayer et al. 2004), AJ890255 – AJ890282 (Kurmayer & Gumpenberger 2006), EU266180 - EU266303 (Christiansen et al. 2006), GQ917053 – GQ917107 (this study)

| Strain No. | Origin | PC-IGS (211 bp) | *mcy*B (1451 bp) | *aer*B  (343 bp) | *apn*C  (470 bp) |
| --- | --- | --- | --- | --- | --- |
| *Planktothrix rubescens* |  |  |  |  |  |
| No34 | Ammersee, DE | AJ558156 | AJ890260 | + | + |
| No81 | - ´´ - | + | AJ890261 | + | + |
| No82 | - ´´ - | EU266279 | AJ749279 | + | + |
| No83 | - ´´ - | EU266280 | AJ749280 | + | + |
| No86 | - ´´ - | + | AJ890262 | + | + |
| No102 | - ´´ - | + | + | GQ917084 | + |
| No103 | - ´´ - | + | + | + | + |
| No12 | Irrsee, AT | + | + | + | + |
| No62 | - ´´ - | AJ558153 | AJ890265 | + | + |
| No65 | - ´´ - | + | AJ890266 | GQ917106 | GQ917065 |
| No87 | - ´´ - | + | + | + | GQ917066 |
| No94 | - ´´ - | + | + | + | + |
| No95 | - ´´ - | + | + | + | + |
| No108 | - ´´ - | EU266281 | AJ749281 | + | + |
| No3 | Mondsee, AT | EU266275 | AJ749276 | GQ917100 | GQ917056 |
| No6 | - ´´ - | + | + | + | + |
| No7 | - ´´ - | + | + | GQ917083 | GQ917053 |
| No8 | - ´´ - | + | + | + | + |
| No17 | - ´´ - | + | + | GQ917097 | GQ917058 |
| No91/1 | - ´´ - | EU266292 | AJ890264 | + | + |
| No97 | - ´´ - | EU266300 | + | + | + |
| No99 | - ´´ - | + | AJ890256 | + | + |
| No100 | - ´´ - | + | + | + | + |
| No106 | - ´´ - | + | + | + | + |
| No111 | - ´´ - | EU266276 | AJ749282 | + | GQ917067 |
| No13 | Schwarzensee, AT | + | AJ890276 | GQ917088 | GQ917057 |
| No14 | - ´´ - | + | + | + | + |
| No18 | - ´´ - | + | AJ890277 | + | + |
| No59 | - ´´ - | + | AJ890278 | GQ917085 | + |
| No60 | - ´´ - | + | AJ890279 | + | + |
| No61 | - ´´ - | + | + | + | + |
| No80 | - ´´ - | EU266269 | AJ749278 | + | + |
| No29/3 | Wörthersee, AT | AJ558148 | + | GQ917099 | GQ917060 |
| No42 | - ´´ - | + | AJ890268 | GQ917103 | GQ917063 |
| No64 | - ´´ - | EU266267 | AJ749277 | + | GQ917054 |
| No77 | - ´´ - | + | AJ890280 | + | + |
| No89 | - ´´ - | + | AJ890281 | + | + |
| No67 | - ´´ - | EU266291 | AJ890273 | + | + |
| No46 | Zürichsee, CH | AJ558151 | AJ890272 | + | GQ917064 |
| No72 | - ´´ - | + | AJ890267 | + | + |
| No75 | - ´´ - | + | AJ890274 | + | + |
| No21/1 | Figur, Vienna, AT | AJ558157 | AJ863131 | GQ917098 | GQ917059 |
| CYA128 | L. Vesijärvi, FI | AJ558144 | + | GQ917087 | + |
| SAG5.89 | Zürich, Limmat, CH | AJ558146 | + | GQ917104 | GQ917081 |
| PCC7821 | L. Gjersjøen, NO | AJ558154 | AJ749283 | + | GQ917080 |
| CCAP1459/14 | Loughrigg Tarn, UK | EU266287 | AJ863132 | GQ917090 | GQ917070 |
| CCAP1459/24 | L. Ören, SE | AJ558150 | AJ890271 | + | GQ917073 |
| CCAP1459/30 | Plöner See, DE | EU266283 | AJ749284 | GQ917093 | GQ917074 |
| CCAP1459/38 | L. Windermere, UK | AJ558149 | + | + | GQ917077 |
| *Planktothrix agardhii* |  |  |  |  |  |
| No41 | Jägerteich, AT | EU266259 | - | - | + |
| No63 | - ´´ - | EU266260 | - | - | + |
| No66 | - ´´ - | EU266261 | - | - | + |
| No28/2 | Wannsee, DE | AJ558143 | - | + | - |
| No31/1 | - ´´ - | EU266265 | AJ749267 | + | + |
| No32 | - ´´ - | EU266277 | AJ749268 | GQ917101 | GQ917061 |
| No39 | - ´´ - | EU266266 | AJ749269 | GQ917102 | GQ917062 |
| CYA126/8 | L. Langsjön, FI | EU266274 | AJ441056 | GQ917086 | GQ917068 |
| PH22 | Lake Bagsværd Sø, DK | EU266258 | - | - | - |
| SAG6.89 | Plußsee, Plön, DE | EU266278 | AJ749271 | GQ917105 | GQ917082 |
| SAG5.81 | Kiessee, Göttingen, DE | EU266257 | - | - | - |
| PCC7805 | Veluwermeer, NL | EU266263 | - | GQ917107 | - |
| PCC7811 | Tassigny, FR | EU266264 | - | - | GQ917079 |
| CCAP1459/11A | L. Windermere, UK | EU266271 | AJ749272 | GQ917089 | GQ917069 |
| CCAP1459/15 | Lough Neagh, N. Ireland, UK | EU266262 | - | - | - |
| CCAP1459/16 | Blelham Tarn, UK | EU266285 | AJ749273 | GQ917091 | GQ917055 |
| CCAP1459/17 | - ´´ - | EU266288 | AJ863133 | + | GQ917071 |
| CCAP1459/21 | Esthwaite Water | EU266284 | AJ749274 | GQ917092 | GQ917072 |
| CCAP1459/23 | - ´´ - | + | + | + | + |
| CCAP1459/36 | L. Gjersjoen | EU266272 | + | GQ917095 | GQ917076 |
| CCAP1459/31 | White Lough, N. Ireland | EU266289 | AJ863134 | GQ917094 | GQ917075 |
| CCAP1460/5 | L. Kasumigaura, Japan | EU266273 | AJ749275 | GQ917096 | GQ917078 |

^+^ expected size of PCR product obtained (not sequenced); - gene fragment not detected

Suppl. Fig. 1: Comparison of the total *Planktothrix* spp. biovolume (mm^3^ L^-1^) estimated from lake water samples (Table 1) by counting under the microscope and by qPCR, mean ± 1 SE. If error bars are not visible, they are hidden behind the symbols. For details on the regression curve see text. The lake samples were classified into red pigmented *Planktothrix* (L. Mondsee, Irrsee, other lakes), green pigmented *Planktothrix* (L. Wannsee, L. Frederiksborg Slotssø, other lakes) and mixed pigmented *Planktothrix*.
